# Acute Severe Aortic Regurgitation From Catastrophic Pannus Obstruction in a Patient With Mechanical Aortic Valve Replacement

**DOI:** 10.7759/cureus.27198

**Published:** 2022-07-24

**Authors:** Ankur Panchal, Ramzi Khalil, Georgios Lygouris, Robert Biederman, Andreas Kyvernitakis

**Affiliations:** 1 Cardiovascular disease, Bassett Medical Center, Cooperstown, USA; 2 Cardiovascular Disease, Allegheny Health Network, Pittsburgh, USA; 3 Cardiovascular Disease, UnityPoint Health, Cedar Rapids, USA

**Keywords:** valvular heart disease, prosthetic valve pannus, aortic regurgitation, prosthetic valve obstruction, mechanical aortic valve

## Abstract

A 69-year-old woman with a mechanical aortic valve presented with decompensated heart failure. Emergent echocardiogram and fluoroscopy demonstrated acute aortic regurgitation due to a dysfunctional mechanical aortic valve and non-obstructive coronary disease. An emergent valve replacement was performed confirming a fixed-open valve with pathology demonstrating obstructive pannus formation without thrombosis or vegetation.

## Introduction

Mechanical valve prostheses, despite having the major limitation of chronic systemic anticoagulation and the growing popularity of bioprosthetic valves, are still commonly performed, especially in young patients with valvulopathies. Major complications of mechanical valves include bleeding risk, endocarditis, valve obstruction, and valve thrombosis [1]. Thrombosis, most common, is usually secondary to underlying cardiac dysfunction, presence of atrial fibrillation, increased thrombogenicity due to valve material, and sub-therapeutic anticoagulation [2]. Prosthetic valve endocarditis, especially early, accounts for approximately 20% of all infective endocarditis cases and requires a prolonged antibiotic course and often surgical intervention [3]. Herein, we present a rare case of acute, fulminant aortic regurgitation due to pannus formation after remote mechanical aortic valve replacement surgically and pathologically not to have been either thrombus or vegetation.

## Case presentation

**Figure 1:**
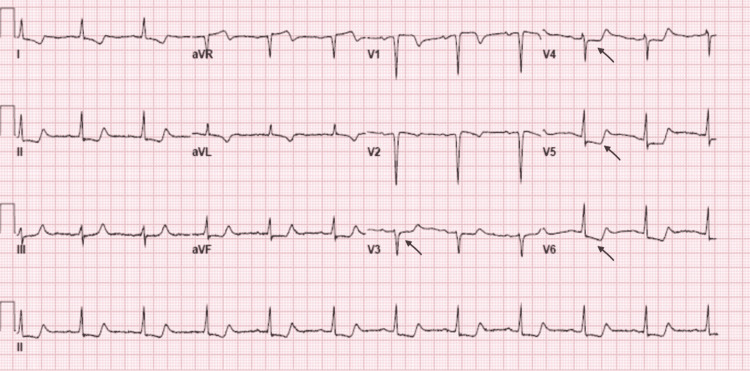
12-Lead EKG showing ST depression in precordial leads (black arrows).

**Figure 2:**
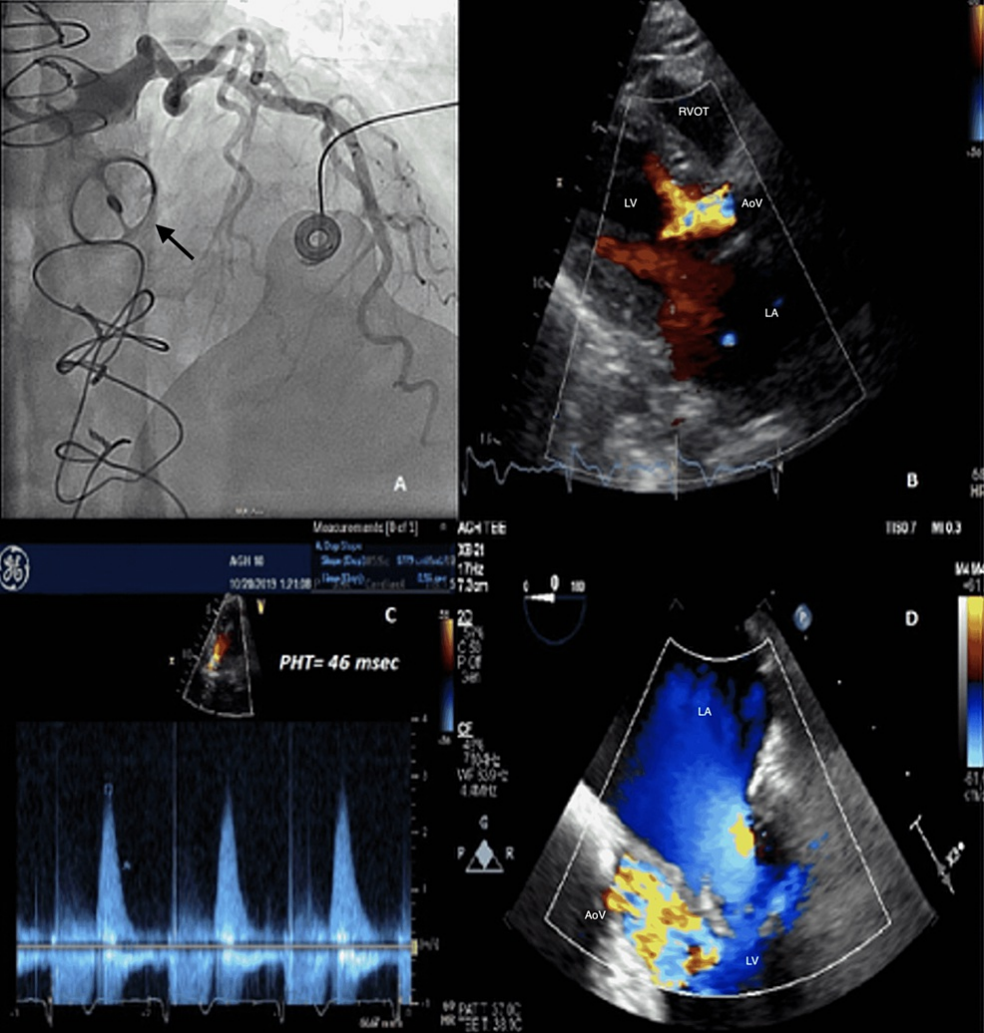
(A) Coronary angiogram revealing normal left coronary artery and immobile tilting disc mechanical aortic valve (black arrow). (B) Para-sternal long axis TTE image with broad-based early diastolic aortic insufficiency. (C) Pressure half time (PHT) of 46 milliseconds indicating severe aortic insufficiency. (D) Three chamber TEE image showing severe aortic insufficiency during early diastole.

A 69-year-old woman with a history of bicuspid aortic valve with mechanical aortic valve replacement (AVR) in 1997 at another institution for bicuspid aortic valve stenosis, and paroxysmal atrial fibrillation presented to the hospital with acute onset of chest pain and dyspnea. On presentation, her blood pressure was 94/73 mmHg, heart rate 126 bpm with a regular rhythm, and required noninvasive ventilation at 100% FiO_2_ for respiratory distress. She had prominent jugular venous distension, bibasilar crackles, and cool extremities. Chest x-ray revealed diffuse bilateral airspace opacities. Labs were significant for Pro-BNP of 1,635 pg/mL, troponin T 0.43 ng/mL, lactate 3.7 mmol/L, and INR of 2.98 without recent fluctuations (Table 1). Her EKG showed ST-segment depressions in the precordial leads (Figure 1). A chest CT angiogram was negative for aortic dissection but showed extensive bilateral ground-glass opacities with interlobular septal thickening. A bedside transthoracic echocardiogram (TTE) demonstrated mild global left ventricular systolic dysfunction, moderate mitral regurgitation, moderate-to-severe tricuspid regurgitation, and brief aortic regurgitation (AR) with suspiciously short, early diastolic pressure half-time of 46 msec along with a dense, rapidly peaking jet via continuous-wave Doppler (Figures 2A-2D). Her previous echocardiogram was 16 months prior and revealed a non-obstructive prosthesis with trivial regurgitation suggestive of washing jets. Due to rapidly deteriorating cardiogenic shock, she required vasopressors, non-invasive ventilation, and intravenous diuretics. She underwent emergent coronary angiography which revealed no evidence of obstructive coronary artery disease, but rapid fluoroscopy was diagnostic of a fixed-open mechanical bileaflet aortic valve prosthesis. Additionally, it showed a rapidly clearing contrast rim along the aortic cusp very suspicious for torrential AR.

**Table 1:** Pertinent lab results of the patient upon presentation

Lab	Patient’s result	Normal range
Pro-BNP (Pro – B type natriuretic peptide)	1635 pg/mL	<125 pg/mL
Troponin T	0.43 ng/mL	0-0.01 ng/mL
Lactate	3.7 mmol/L	0.5-2.2 mmol/L
INR (International normalized ratio)	2.98	<1.1

Urgent consultation for cardiothoracic surgery was made and performed with emergent redo-sternotomy while the mechanical aortic valve was replaced after an intraoperative trans-esophageal echocardiogram (TEE) confirmed severe, torrential (4+) aortic regurgitation and an immobile, fixed-open mechanical aortic valve. On careful surgical exploration, there was large, irregular, and expansive pannus formation on the ventricular side of the valve, which was sent to pathology confirming pannus formation without thrombus. Importantly, the TTE and TEE were corroborated as there was no evidence visually or on histopathology of thrombus or, via microbiology, for vegetation. A 21-mm bioprosthetic aortic valve was eventually placed with near-normal intra and postoperative gradients. The patient tolerated the extremis of her presentation well and was subsequently discharged to inpatient rehabilitation.

Post-operatively, the patient had a non-unexpected complex hospitalization. While she was successfully weaned off veno-arterial extracorporeal membrane oxygenation (VA ECMO) on postoperative day 1, she had a prolonged course in the hospital requiring management of biventricular heart failure and was eventually discharged on day 30.

## Discussion

Mechanical prosthetic valves require lifelong anticoagulation therapy given increased thrombogenicity [1]. Prosthetic valve obstruction (PVO) is a rare and dreaded complication of mechanical prostheses, caused usually by thrombus and uncommonly by pannus formation [1]. The latter is attributed largely due to fibroelastic reactions from the surrounding tissues involving multiple signaling proteins, including TGF-βeta, which results in fibrinous and collagenous growth, especially around the annulus of the prosthetic valve [1]. PVO from pannus formation usually, unlike, in this case, causes gradual valvular stenosis, and patients typically have a remote history of valve replacement with evidence of therapeutic anticoagulation as compared to the PVO caused by thrombus, which is associated with hyperacute presentation and valvular dysfunction. Surgical replacement of the valve is usually indicated in the majority of patients due to the risk of systemic embolization and acute decompensation [4,5]. When PVO is suspected, non-invasive modalities such as TTE, TEE, gated computed tomography, and cinefluoroscopy can confirm the diagnosis [1,6]. Cardiac MRI due to the para-magnetic artifact of the metallic valve is not a technique of choice [7].

Our case illustrates a common clinical presentation with a broad differential diagnosis and reveals that acute mechanical valve dysfunction can occur due to pannus formation despite consistent therapeutic anticoagulation. Her aortic valve prosthesis had been without incidence for over 23 years. Indeed, despite adequate anticoagulation, the process of gradual fibrin deposition and collagen formation is an inevitable sequela resulting in gradual pannus only accelerated with non-compliant anticoagulation use. Nevertheless, within two hours of presentation, our patient required non-invasive ventilation with 100 % FiO_2_ and multiple vasopressor medications for hypotension. Consistent imaging parameters raised concerns of acute aortic regurgitation due to low-pressure half-time but was consistent with a brief, early diastolic jet uniquely discordant from her auscultation. It is a clinical pearl that severe, acute AR may be markedly underestimated by physical examination due to brief, inappreciable murmur and by TTE due to poor LV compliance along with rapid diastolic Ao-LV pressure equalization leading to brief color Doppler flow. In such situations, since LV compliance does not immediately change, grading of AR to include PHT can greatly underestimate the estimation of AR [8]. As commonly seen in these situations, a dedicated TEE could not be performed due to rapidly evolving hemodynamic instability. Cinefluoroscopy however quickly confirmed our suspicions. Ellensen et al. reviewed >1,000 patients with mechanical valve prostheses and found an incidence of acute obstruction from pannus formation being 0.7/1,000 with a median time-to-prosthetic dysfunction of 11 years. Interestingly, women and younger patients had a higher risk for pannus formation [9]. Unfortunately, considering a remote history of valve replacement in this patient, we were not able to gather data on valve type and size. 

The initial step for diagnosis of PVO, following careful physical examination in forward/prone position, usually involves TTE with Doppler studies which provide visualization of the valve and quantification of the severity of valve dysfunction [1,6,10]. However, the visualization and Doppler assessment of mechanical prosthesis in the aortic position can be limited by TTE due to acoustic shadowing [11]. In such cases, TEE can usually differentiate thrombus from pannus (smaller and more echodense without mobility) helping to guide rapid clinical decision-making [6,11]. Prompt surgical replacement is usually indicated in these patients, especially in the presence of hemodynamic instability which is associated with exceptionally high mortality.

## Conclusions

This case highlights a delayed but hyperacute presentation of mechanical valve obstruction by pannus formation, in a compliant patient with stable therapeutic INR. Acute, severe AR is poorly tolerated in an otherwise non-remodeled left ventricle and is often underestimated by both physical examination and echocardiography. A very high index of clinical suspicion in such patients is required due to rapid clinical deterioration but can often result in remarkable salvage in otherwise deadly circumstances.
